# Consensus Guidelines for Teledermatology: Scoping Review

**DOI:** 10.2196/46121

**Published:** 2023-05-15

**Authors:** Mollie R Cummins, Triton Ong, Julia Ivanova, Janelle F Barrera, Hattie Wilczewski, Hiral Soni, Brandon M Welch, Brian E Bunnell

**Affiliations:** 1 College of Nursing University of Utah Salt Lake City, UT United States; 2 Department of Biomedical Informatics Spencer Fox Eccles School of Medicine University of Utah Salt Lake City, UT United States; 3 Doxy.me, Inc Rochester, NY United States; 4 Department of Psychiatry and Behavioral Neurosciences University of South Florida Tampa, FL United States; 5 Biomedical Informatics Center Public Health and Sciences Medical University of South Carolina Charleston, SC United States

**Keywords:** COVID-19, dermatology, teledermatology, telehealth, telemedicine, consensus guidelines, guidelines, recommendations

## Abstract

**Background:**

Consensus guidelines and recommendations play an important role in fostering quality, safety, and best practices, as they represent an expert interpretation of the biomedical literature and its application to practice. However, it is unclear whether the recent collective experience of implementing telemedicine and the concurrent growth in the evidence base for teledermatology have resulted in more robust guidance.

**Objective:**

The objective of this review was to describe the extent and nature of currently available guidance, defined as consensus guidelines and recommendations available for telemedicine in dermatology, with guidance defined as consensus or evidence-based guidelines, protocols, or recommendations.

**Methods:**

We conducted a single-reviewer scoping review of the literature to assess the extent and nature of available guidance, consensus guidelines, or recommendations related to teledermatology. We limited the review to published material in English since 2013, reflecting approximately the past 10 years. We conducted the review in November and December of the year 2022.

**Results:**

We identified 839 potentially eligible publications, with 9 additional records identified through organizational websites. A total of 15 publications met the inclusion and exclusion criteria. The guidelines focused on varied topics and populations about dermatology and skin diseases. However, the most frequent focus was general dermatology (8/15, 53%). Approximately half of the telemedicine guidance described in the publications was specific to dermatology practice in the context of the COVID-19 pandemic. The publications were largely published in or after the year 2020 (13/15, 87%). Geographical origin spanned several different nations, including Australia, the United States, European countries, and India.

**Conclusions:**

We found an increase in COVID-19–specific teledermatology guidance during 2020, in addition to general teledermatology guidance during the period of the study. Primary sources of general teledermatology guidance reported in the biomedical literature are the University of Queensland’s Centre for Online Health and Australasian College of Dermatologists E-Health Committee, and the American Telemedicine Association. There is strong evidence of international engagement and interest. Despite the recent increase in research reports related to telemedicine, there is a relative lack of new guidance based on COVID-19 lessons and innovations. There is a need to review recent evidence and update existing recommendations. Additionally, there is a need for guidance that addresses emerging technologies.

## Introduction

The use of telemedicine in dermatology practice dates to the mid-1990s when early innovators recognized it as a promising means of delivering dermatology specialty care to remote and underserved populations [1]. However, “teledermatology” lacked widespread adoption before the COVID-19 pandemic due to policies restricting practice and negatively affecting teledermatology services reimbursement. In 2020, the public health measures and policy changes triggered by the COVID-19 pandemic led to considerable growth in the adoption of telemedicine. The regulatory changes related to telemedicine that occurred in the United States during 2020 are summarized elsewhere [2] and include important changes in Centers for Medicare & Medicaid Services policies related to interstate licensure, reimbursement, and Health Insurance Portability and Accountability Act of 1996 encryption requirements. Empowered by these regulatory changes, individuals and groups quickly adopted telemedicine to deliver patient care, employing the best available methods and models or none, out of sheer necessity.

A bolus of telemedicine-focused reports in the biomedical literature accompanied widespread and dramatic increases in the adoption of telemedicine during 2020. In the biomedical literature database PubMed [3], the number of records containing the keyword “telemedicine” in 2020 and 2021 is approximately double the number in 2019, with over 8000 records per year. The array of digital health technologies available to support telemedicine delivery has also continued to mature, with the widespread availability of biosensors and communication platforms (eg, SMS text messaging platforms, chatbots, and mobile apps) and smartphone imaging, alongside transformative advancements in artificial intelligence. Many recent reports describe applications of these rapidly developing technologies in dermatology [4-9]. Attention is turning to quality, safety, and best practices in a sustained health care delivery model that incorporates telemedicine in a rapidly evolving landscape of digital health technologies.

Consensus guidelines and recommendations play an important role in fostering quality, safety, and best practices, as they represent an expert interpretation of the biomedical literature and its application to practice. However, it is unclear whether the recent collective experience of implementing telemedicine and the concurrent growth in the evidence base for teledermatology have resulted in more robust guidance. The objective of this review was to describe the extent and nature of currently available guidance, defined as consensus guidelines and recommendations, available for the practice of telemedicine in dermatology, with guidance defined as consensus- or evidence-based guidelines, protocols, or recommendations.

## Methods

### Overview

We conducted a single-reviewer scoping review of the literature to assess the extent and nature of available guidance, consensus guidelines, or recommendations related to the use of telemedicine in dermatology practice. Here, we define telemedicine according to the Health Resources and Services Administration of the US Department of Health and Human Services [10] definition as “the use of electronic information and telecommunications technologies to support and promote long-distance clinical health care, patient and professional health-related education, public health, and health administration.” According to the US Department of Health and Human Services, these technologies include “videoconferencing, the internet, store-and-forward (SAF) imaging, streaming media, and terrestrial and wireless communications” [10]. Before initiating the review, we searched 6 sources for existing protocols or reviews on this subject and found none. Sources searched on November 21, 2022, included PROSPERO [11], Epistemonikos [12], Cochrane Library [13], and CINAHL Complete (EBSCOhost) [14]. One closely related review is that recently published by Dovigi et al [15], which focuses on quality assessment.

We conducted the review according to guidance from the latest *JBI Manual for Evidence Synthesis* [16]. Specifically, we followed the process of a scoping review with Arksey’s five stages: (1) identifying the research question; (2) identifying relevant studies; (3) study selection; (4) charting the data; and (5) collating, summarizing, and reporting the results [17]. However, we streamlined and expedited the review process by using a single reviewer to screen and code publications. We used EndNote (Clarivate Analytics) to manage and deduplicate citations. We used Covidence (Veritas Health Innovation) to further deduplicate, screen, and select studies and to perform data extraction.

### Literature Search

We searched multiple web-based databases: Cochrane Library, Scopus, PubMed, Epistemonikos, Cochrane Library, and CINAHL Complete (EBSCOhost). We used keywords and controlled subject headings unique to each database and detailed in Multimedia Appendix 1, designed to identify terms that included telehealth, telemedicine, teledermatology, dermatology, guidelines, and recommendations. We excluded the ECRI Guidelines Trust, as it was publicly unavailable during the review. We also examined materials found on the following federal and organizational websites: the American Telemedicine Association, the American Academy of Dermatology, the American Dermatological Association, and the US Agency for Healthcare Research and Quality. We summarize the search strategy and results in Multimedia Appendix 1. We limited the review to published material in the English language, published since 2013, reflecting approximately the past 10 years. We conducted the review in November and December 2022.

### Article Selection (Eligibility Criteria)

The eligibility criteria for article selection are listed in Textbox 1.

**Eligibility criteria for article selection.**

**Inclusion criteria:**

• We included reports of consensus-based practice guidelines or aggregated sets of recommendations related to dermatology using telehealth or telemedicine, published since January 1, 2013, and originating from any country.

• We include reports that present guidelines published separately in a more comprehensive format, consistent with this commonly encountered reporting pattern for guidelines and recommendations.

**Exclusion criteria:**

• We excluded reports without a primary focus on dermatology or dermatological conditions and guidelines that lack specific telehealth or telemedicine practice recommendations.

• We also excluded guidance not based on a consensus process or study. Additionally, we excluded material not available in the English language.

### Assessment, Extraction, and Analysis

We did not conduct a formal quality assessment of underlying studies because consensus guidelines constitute an evaluation and recommended application of evidence by experts. Our goal was to map available consensus guidance rapidly. A single reviewer extracted variables (Table 1) describing the characteristics of the publications using Covidence. We conducted an initial manual data review to identify and resolve any needs for categorization or standardization of nomenclature. We conducted frequency analysis to describe the type and distribution of variables as presented in Table 1 and provide a summary list of articles, guidelines, and their characteristics (Table 2).

**Table 1:** Descriptive summary of publications.

Authors	Years	Guideline described	Brief description of the publication
Abbott and Soyer [18]	2020	A CLOSE-UP guide to capturing clinical images	Supplement to the Australian teledermatology guidelines; presents an acronym that guides capture of clinical images.
Abbott et al [19]	2020	Practice guidelines for teledermatology in Australia	Presents a review of the literature on which the guidelines were based.
Abbott et al [20]	2020	Practice guidelines for teledermatology in Australia	Guidelines for teledermatology for the Australian context, developed by The University of Queensland's Centre for Online Health in collaboration with The Australasian College of Dermatologists E-Health Committee.
Arruda et al [21]	2020	Recommendations for Dermatology Office Reopening in the Era of COVID-19	“A group of international experts was assembled to formulate guidance and best-practices for resuming dermatology practices in a COVID-19 era” [21].
Belinchón et al [22]	2020	Managing psoriasis consultations during the COVID-19 pandemic: recommendations from the Psoriasis Group of the Spanish Academy of Dermatology and Venereology	Statement of recommendations to guide dermatologists “who treat psoriasis, especially in cases where patients are receiving treatment or are about to initiate treatment with selective immunomodulators or immunosuppressants” [22].
Brochez et al [23]	2020	Recommendations for skin cancer consultation and surgery during COVID-19 pandemic	“Recommendations developed by the Belgian Association of Dermato-Oncology for prioritization of patients in the field of dermato-oncology during COVID-19 pandemic.” [23]
Chatterjee and Das [24]	2021	Management of vitiligo amidst the COVID-19 pandemic: a survey and resulting consensus	Survey of experts re: appropriate management of vitiligo during the COVID-19 pandemic.
Deda et al [25]	2022	Dermoscopy practice guidelines for use in telemedicine	Summary of American Telemedicine Association teledermoscopy guidelines.
de Vere Hunt et al [26]	2021	Telehealth for older adults with skin disease: a qualitative exploration of dermatologists’ experiences and recommendations for improving care	Recommendations for use of telehealth with older adults based on qualitative interviews with a sample of dermatologists.
Finnane et al [27]	2017	ISIC recommendations for imaging standardization	Article “translates” ISIC recommendations for imaging standardization into clinical application [27].
Frieden et al [28]	2020	Management of infantile hemangiomas during the COVID pandemic	“The Hemangioma Investigator Group has created consensus recommendations for management of IH [infantile hemangioma] through telemedicine” [28].
McKoy et al [29]	2016	American Telemedicine Association Teledermatology Practice Guidelines	Practice guidelines for teledermatology.
Micali et al [30]	2020	The Italian dermatologic community facing COVID-19 pandemic: recommendation from the Italian Society of Dermatology and Venereology	Emergency plan for dermatology practice during the COVID-19 pandemic.
Stoff et al [31]	2020	Guiding principles for prioritization of limited in-person dermatology appointments during the COVID-19 pandemic	Guiding principles for allocating in-person dermatology appointments during COVID-19.
Zic et al [32]	2020	United States cutaneous lymphoma consortium recommendations for treatment of cutaneous lymphomas during the COVID-19 pandemic	US consortium recommends strategies for treating cutaneous lymphomas during the COVID-19 pandemic.

**Table 2:** Characteristics of included reports.

Variables			Frequency				Reports, %		
			Absolute		Relative				
**Year published**									
	2016	1		0.07		7			
	2017	1		0.07		7			
	2020	10		0.67		67			
	2021	2		0.13		13			
	2022	1		0.07		7			
	Total	15		1		100			
**Country of origin**									
	Australia	4		0.27		27			
	Belgium	1		0.07		7			
	India	1		0.07		7			
	International (multiple)	2		0.13		13			
	Italy	1		0.07		7			
	Spain	1		0.07		7			
	United States	5		0.33		33			
	Total	15		1		100			
**COVID-19–specific guidance?**									
	Yes	7		0.47		47			
	No	8		0.53		53			
	Total	15		1		100			
**Focus**									
	Dermato-oncology	2		0.13		13			
	General	8		0.53		53			
	Infantile hemangioma	1		0.07		7			
	Older adults	1		0.07		7			
	Psoriasis	1		0.07		7			
	Vitiligo	1		0.07		7			
	Total	15		1		100			
**Consensus group**									
	Professional society	11		0.73		73			
	Author-assembled panel	4		0.27		27			
	Total	15		1		100			
**Nature of guidance**									
	Guideline	7		0.47		47			
	Recommendation	8		0.53		53			
	Total	15		1		100			

## Results

### Screening and Selection

**Figure 1:**
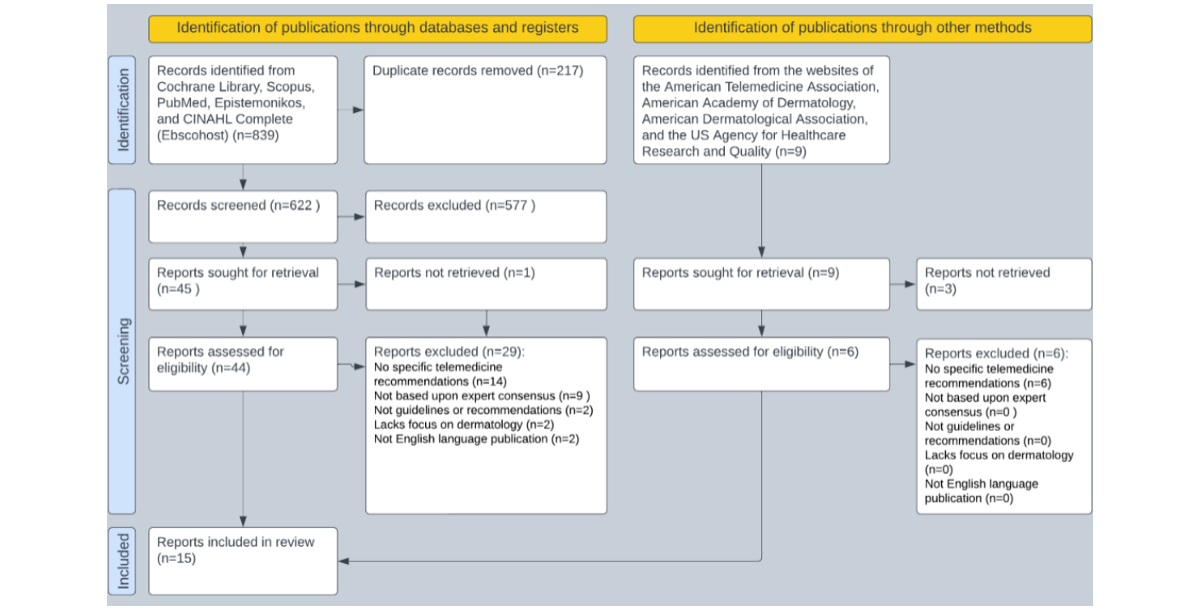
Preferred Reporting Items for Systematic Reviews and Meta-Analyses (PRISMA) diagram of the review process.

We summarize the search, screening, and selection process results in Figure 1. The biomedical literature search process identified 839 potentially eligible publications, with an additional 9 records identified through organizational websites. After deduplicating search results, we manually screened the titles and abstracts of 622 records for inclusion, followed by 54 full-text reviews (both biomedical literature and publications retrieved from organizational websites), to verify possibly eligible reports. After we completed screening and full-text review, 15 publications met the inclusion and exclusion criteria (Table 1). We provide a list of all the reports included in Table 1.

### Publication Characteristics

**Figure 2:**
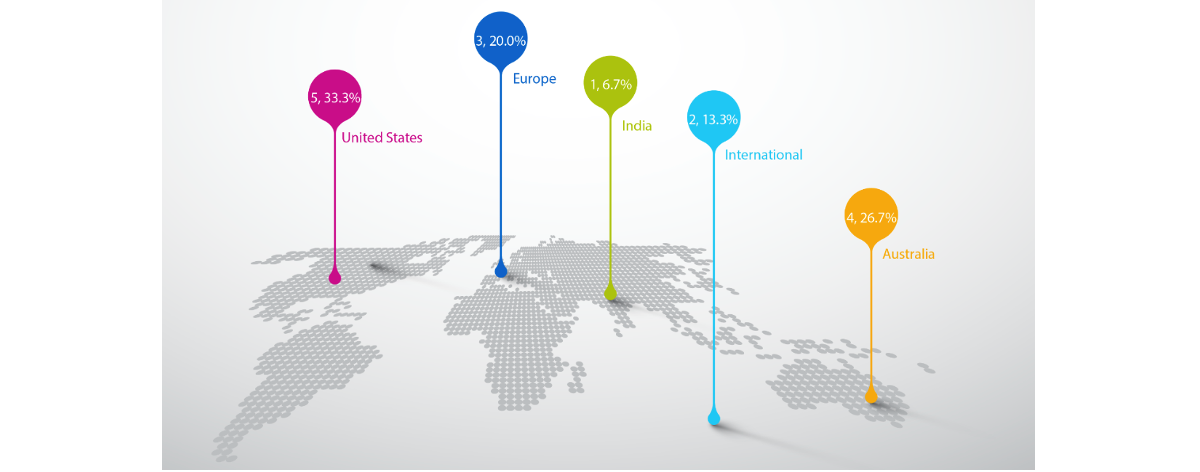
Frequency and percentage of report origin.

We present publication characteristics (Tables 1 and 2 and Figure 2). The included items were primarily published in or after the year 2020 (13/15, 87%). The origin of reports spans several different nations, including Australia, the United States, European countries, and India (Figure 2). Approximately half of the guidance consisted of guidelines (7/15, 47%), with the remaining guidance presenting more general recommendations (8/15, 53%). Also, about half of the telemedicine guidance described in the publications was specific to dermatology practice in the context of the COVID-19 pandemic (7/15, 47%).

In most cases, the source of the guidance was a professional organization or society (11/15, 73%) rather than an independently assembled sample or panel. The guidelines focused on varied topics and populations (Tables 1 and 2) related to dermatology and skin diseases. However, the most frequent focus was general dermatology (8/15, 53%). We briefly describe each report and the guidelines described in each publication (Table 1). Given multiple guidelines addressing common imaging aspects, we present a summary comparison of recommendations in Table 3.

**Table 3:** Imaging-focused recommendations.

	Abbott and Soyer [18]	Abbott et al [20]	Deda et al^a^ [25]	Finnane et al [27]	McKoy et al [33]
Preparation	Obtain consent	Obtain consent Remove jewelry and clothing	Apply liquid or gel to the skin	Remove jewelry	Avoid jewelry and clothing Use chaperone or legal guardian if appropriate Clean skin with alcohol pad
Lighting	Maximize natural light Use overhead light with flash	Use flash	—^b^	Natural light is best Broad spectrum lighting Avoid flash Position light oblique to skin surface	Minimal background lighting Diffuse, indirect Additional fluorescent or full-spectrum lighting may be needed Use flash in case of shadow
Positioning, framing, and orientation	Position patient to optimize image accuracy Center lesion in frame Overview, mid-range, and close-up images	Camera position perpendicular to skin surface Identification markers adjacent to lesion Center lesion in frame Overview, mid-range, and close-up images Begin and end with a photograph of identifying information	Consistent orientation across images Inclusion of anatomical sites in regional images Place camera perpendicular to skin surface	Center lesion in frame Close-up images should include lesion plus equal area of surrounding skin Multiple close-up images if needed for large lesions Consistent orientation across images Cephalic orientation preferred	Overview, mid-range, and close-up images Camera perpendicular to skin Center lesion in frame Use identification markers
Measurement	—	Dermoscopic images should include sizing	Inclusion of diameter scale	Include digital or physical ruler. Place ruler with same orientation as camera	Use measurement tools as appropriate; include a ruler in dermoscopy images
Background	Neutral blue or gray	Solid, neutral	—	Solid Color dependent upon skin color; black for lighter skin; sky blue for darker skin	Solid, neutral, and nonreflective
Resolution	—	Minimum resolution, consistent settings	Sufficient resolution for regional and close-up images with file size of at least 200 KB Digital scales (integrated with device) preferred to physical scales Place scale with same orientation as the dermatoscope	—	Minimum resolution of 1024 × 768 pixels
Focus or field	—	—	Deep depth of field Manually or automatically focus image	—	Use macro mode Use autofocus
Color	—	—	Image color resolution of 24 bits	Periodically calibrate equipment to prevent changes	Calibrate color and white balance
Process	Carefully evaluate image quality with attention to focus, overexposure, representative color Recapture images if necessary	Review images for quality before sending them to a dermatologist Review of images by a dermatologist on appropriate or newer display using review software	—	—	—
Image management	Delete from photography device after uploading to patient’s file Include narrative of clinical context with photos	Secure image storage as part of medical record	Image transmission, processing, and storage according to DICOM (Digital Imaging and Communications in Medicine) standard	Images should be stored May require manual link to patient record Storage according to DICOM	Images become part of the medical record
Dermoscopy considerations	—	Consider whether both polarized and nonpolarized dermoscopy images are appropriate	Polarized vs nonpolarized lighting at discretion of clinician, but generally at least one polarized image Nonpolarized light under specific circumstances	Use of polarized vs nonpolarized at discretion of clinician, dependent upon lesion Generally, at least one polarized image Polarized light for blood vessels, red areas, shiny white lines or clods or rosettes Nonpolarized light for structures such as milia cysts	—
Videography	—	—	—	—	Freeze-frame capture is useful Gradual movement of a video camera for overview, mid-range, and close-up images

^a^The recommendations by Deda and colleagues [25] pertain entirely to dermoscopy; the recommendations by McKoy and colleagues [29] encompass both synchronous and asynchronous imaging.

^b^Not available.

## Discussion

### Principal Results

We conducted a single-reviewer scoping review to assess available guidance for the practice of teledermatology. We identified 15 reports describing 13 unique guidelines or sets of recommendations. We conducted our analysis based on publications, as in some cases, the publications described different aspects of guidelines that were not otherwise available. Professional societies or organizations created most guidance, and the guidance addressed the management of multiple specific skin diseases, in addition to general dermatology.

We found that most publications were published during or after 2020, the onset of the COVID-19 pandemic. Approximately half of the publications contained guidance specific to the circumstances of the COVID-19 pandemic, which included shortages of personal protective equipment, quarantine, and public health measures that had lockdowns [34]. For example, the report by Belinchón et al [22] provides recommendations for managing psoriasis in the context of COVID-19 amidst health considerations and Italy's public health measures. Specifically, they recommended that a consistent clinician supervise care delivery across in-person and telemedicine encounters and alternating in-person and telemedicine visits. The Belgian Association of Dermato-Oncology similarly reported guidance on prioritizing patients for skin cancer consultation and surgery, with direction to simply consider teleconsultation when feasible [23].

The remaining part of the reports pertained to the use of telemedicine in dermatology, independent of pandemic circumstances. The two primary sources of general teledermatology guidance discovered in this review were as follows: (1) the University of Queensland’s Centre for Online Health and the Australasian College of Dermatologists E-Health Committee (UQ-ACD) [18-20], and (2) the American Telemedicine Association (ATA) [25,29]. Most of the UQ-ACD and ATA guidance was issued before the COVID-19 pandemic. The UQ-ACD guidelines address general dermatology practice in Australia and encompass technology, environment, quality and safety, patient selection, informed consent, and the acquisition and storage of clinical images [20]. There is also companion clinical guidance for capturing clinical images [18]. Although access to the ATA guidelines is limited to those holding organizational memberships, the guidelines are partially described in publicly available reports. ATA guidance consists of practice guidelines for general teledermatology as well as teledermoscopy. The general teledermatology guidelines were originally issued in 2007 and revised in 2016, with teledermoscopy guidelines issued most recently in 2021-2022 (report published in 2022). They are topically comprehensive, encompassing environmental, clinical, and administrative considerations, with specific guidance for imaging [25,29].

Most guidance originated in Australia or the United States. However, our review evidences global engagement in creating guidance for teledermatology, as shown in Figure 2. There is international interest in guidance for teledermatology, despite international variation in payment, infrastructure, health system characteristics, and health priorities. However, only 2 reports described international guidance, one focusing on imaging standards [27] and the other focusing on reopening clinics during the COVID-19 pandemic [21].

Given the high recall search strategy, the items excluded during the screening process typically mentioned the keywords but were unrelated to guidelines or recommendations. Others represented literature reviews or systematic reviews of scientific evidence. We excluded 8 reports at the full-text review stage because they were not consensus-based; these were primarily letters to the editor by individuals or small teams; 2 reports were educational or tutorial. For example, Mondal and Mondal [35] presented a tutorial on electronic signatures and document storage for teledermatology practitioners.

### COVID-19–Specific Recommendations

As previously indicated, approximately half of the reports focused on guidance for dermatology practice during the COVID-19 pandemic. These reports focus on emergency plans for providing dermatology care, including triaging patients for in-person and telemedicine visits and highlighting the relevant considerations for integrating telemedicine into practice. For example, the Psoriasis Group of the Spanish Academy of Dermatology and Venereology published recommendations to guide dermatologists who treat psoriasis [22]. Those recommendations indicate that telemedicine visits between a patient and provider may be acceptable. Arruda et al [21] presented international recommendations for reopening dermatology offices, with summary guidance for the successful integration of telemedicine into practice, including SAF consultation, new consultations, and attention to local government regulations. Brochez et al [23] made pragmatic recommendations about triaging the care of dermato-oncology patients and deciding when care can and cannot be postponed. They organized encounter or presentation types into 3 categories: urgent, semiurgent, and low-priority. Chatterjee and Das [24] surveyed expert dermatologists to determine when patients with vitiligo can be appropriately managed via telemedicine.

### Imaging

Imaging is critical for teledermatology practice. The multiple reports and the guidelines they describe address imaging considerations [18,20]. The CLOSE-UP guideline is a particularly useful tool for clinicians photographing lesions to obtain teledermatology consultation using a SAF model [18]. CLOSE-UP addresses the need for informed consent with any image capture and storage. It also guides clinicians in the photography process to use natural light or, overhead lighting with flash against a gray or neutral blue background. This guideline also describes a method of taking a series of photographs, including a wider frame overview image, a mid-range image, and one or more close-up images, all with a consistent orientation. The purpose of a sequence of images is to enable assessment of how lesions are distributed and their location on the body, in addition to the more closely photographed lesions themselves. The CLOSE-UP guidelines encourage the evaluation and recapture of images as necessary, uploading them to a patient’s file, then deleting them from the photography device. It also highlights the importance of providing the teledermatologist with relevant clinical context, in addition to images, including findings that are not evident in the images.

Finnane et al [27] call for standardization of image capture in dermatology and present a series of recommendations developed by the International Skin Imaging Collaboration (ISIC), broadly consistent with CLOSE-UP, but also addressing dermoscopy. Among multiple lighting considerations, they recommend avoiding the use of flash in clinical photography, noting its effects on image contrast, the inclusion of reflections in images, and effects on skin tone. They also address considerations that the teledermatologist should apply in using polarized and nonpolarized lighting in dermoscopy. The ISIC recommendations, like CLOSE-UP, specify an optimal background color. However, ISIC recommends using different background colors for different skin tones, with black for lighter skin and blue for darker skin. ISIC recommends using digital scales, integrated into photographic devices or software, rather than adhesive scales, because applying the adhesive causes some variability and obscures skin and appropriate placement of a ruler can be challenging. ISIC notes the importance of high-resolution images and provides a detailed guide for selecting a resolution. They also provide guidance on color calibration, noting that photography devices must be regularly calibrated. The ISIC guidance on image storage notes that both clinical information and images need to be stored as part of the medical record, and points to the existing and widely adopted DICOM (Digital Imaging Communication in Medicine) standard for doing so.

The ATA guidelines described by Deda et al [25] provide specific guidance for dermoscopy in telemedicine. The scope of the guidelines is consistent with the imaging considerations noted in CLOSE-UP and the ISIC recommendations, but specific to dermoscopy. These guidelines provide indicators of appropriate resolution and lighting, as well as focus or depth of field, field of view, color, and image quality with an easy-to-consume quality checklist and a step-by-step process diagram for photography in the context of SAF consultation. These guidelines also favor using digital scales versus physical scales, high-resolution images, and multiple images with varied field of view but a consistent orientation.

### When is Teledermatology Appropriate?

Multiple reports emphasize the importance of provider expertise, and caution that telemedicine should only be carried out by appropriately credentialed specialists. Further, these reports emphasize the importance of the clinician's judgment in assessing whether teledermatology is appropriate for a given patient. There is less agreement on the specific circumstances and models that should be used. Guidance was frequently focused on particular clinical conditions within general dermatology. However, multiple reports note the various factors to be considered, including whether the patient is new or established, the nature of their presentation, and the role of teledermatology in a more extensive care delivery process with sequenced encounters that can include both in-person and teledermatology visits. Factors that influence the appropriateness of teledermatology include the need for a head-to-toe physical examination, whether the patient is new or under ongoing treatment, and the availability of appropriate tools and environment (eg, dermoscopy, established systems and processes for managing images, teleconsent process, etc). There is an acknowledgment that certain types of encounters, such as initial consultation for cosmetic procedures, can easily be appropriate for teledermatology. One of the major use cases for teledermatology is consultation with referring providers, which is carried out using an established process with more controlled and standardized image capture, and clinical assessment information is captured during an in-person visit with the referring provider, a very different scenario from direct-to-patient assessment. There is a need to ensure that patient expectations regarding their ability to obtain care via telemedicine versus in-person visits are realistic and that they understand that clinical circumstances may warrant a different care modality.

### Recent Evidence

From 2020 to the present, thousands of publications in the biomedical literature focused on aspects of telemedicine and telehealth. Many of these studies were an outgrowth of widespread adoption during the COVID-19 pandemic and the opportunity to study numerous aspects of telemedicine and teledermatology. Among these studies were trials of teledermatology interventions, for example, a trial of teledermatology with psoriasis patients, and pilot studies of teledermatology consultation in novel settings, such as the emergency department and inpatient environments [36-39]. There has been substantial growth in the literature describing the acceptability of teledermatology from the patient and provider perspective across many settings and cultures [40-45]. Technical innovations are also evolving; guidelines and recommendations could address new dermoscopy devices, artificial intelligence, and ultrasonography [46,47]. The pandemic yielded new insights into the process and workflow considerations of implementing teledermatology [48,49]. In effect, there is a substantial amount of recent literature that requires expert review and consideration in updates to existing guidelines. This recent evidence could enable more explicit guidelines for determining the appropriateness of teledermatology.

### Limitations

The primary limitation of this review is that we were unable to discover consensus guidance that exists but has not been reported in the biomedical literature. We surmise that panels of experts have generated guidance for internal use by large health care organizations, but the guidance was not shared externally or reported in the biomedical literature or were not revealed using the search strategy that we employed. Additionally, we could not access some guidance because access was restricted to members. This finding highlights the need for open access to consensus guidance and the importance of communicating about guidance in the biomedical literature so that clinicians from resource-constrained settings can benefit from it. We acknowledge that teledermatology is not frequently used in low- to middle-income countries, and so these geographical areas may be underrepresented in the review.

As a single-reviewer scoping review, this review lacked the benefit of a second reviewer in making determinations during the screening and selection process. However, we chose this approach to expedite the process and ensure timely publication, which is often challenging for structured reviews [50]. Moreover, we adhered to the recommended process and reporting standards for this type of review.

### Conclusions

This single-reviewer scoping review described the extent and nature of currently available teledermatology guidance. We observed a large number of COVID-19–specific guidelines or recommendations during 2020 and fewer reports of general teledermatology guidance. The primary sources of general teledermatology guidance are the UQ-ACD and ATA, and there is strong evidence of international engagement and interest. Given a substantial recent increase in reports of research related to telemedicine, there is relatively little new guidance based on COVID-19 lessons and innovations. There is a need to review recent evidence and update existing recommendations. Additionally, there is a need for guidance that addresses emerging technologies. Open access and public availability are crucial to meet the global demand for quality and safety of teledermatology.

## Appendix

Multimedia Appendix 1 Search strategy.

## Abbreviations

ATA: American Telemedicine Association
ISIC: International Skin Imaging Collaboration
SAF: store-and-forward
UQ-ACD: University of Queensland’s Centre for Online Health and Australasian College of Dermatologists E-Health Committee
