# Supplementary material for: Consensus Guidelines for Teledermatology: Scoping Review
Source: JMIR Dermatol. 2023 May 15;6:e46121. doi: 10.2196/46121 (PMC10335147; doi:10.2196/46121)
Supplement: Multimedia Appendix 1 [file derma_v6i1e46121_app1.docx]

**Appendix 1. Search Strategy**

1. Biomedical Literature

| **Database** | **String** | **Search Date** | **Results** |
| --- | --- | --- | --- |
| ECRI Guidelines Trust (<https://www.ecri.org/solutions/ecri-guidelines-trust>) | Site down as of 11/21/2022 (message that it is unavailable during revision - no date for availability) | 11/21/2022, 11/23/2022, 12/6/2022 | N/A |
| Scopus (scopus.com) | KEY ( derm* ) AND KEY ( tele* ) AND TITLE-ABS-KEY ( guid* OR recommend* ) AND PUBYEAR AFT 2012 | 11/23/2022 | 298 |
| PubMed (pubmed.gov) | ("guid*"[All Fields] OR "recommendation*"[All Fields]) AND "tele*"[All Fields] AND "derm*"[All Fields] AND (2013:2022[pdat]) | 11/21/23/2022 | 452 |
| Epistemonikos (www.epistemonikos.org) | (title:(derm* AND (guid* OR recommend*) AND tele*) OR abstract:(derm* AND (guid* OR recommend*) AND tele*)) limited to 2013-2022 | 11/23/2022 | 61 |
| Cochrane Database of Systematic Reviews (www.cochranelibrary.com) | derm* in Title Abstract Keyword AND tele* in Title Abstract Keyword AND guid* OR recommend* in Title Abstract Keyword - with Cochrane Library publication date Between Jan 2013 and Dec 2022, in Cochrane Reviews, Clinical Answers, Special Collections (Word variations have been searched) | 11/23/2022 | 4 |
| CINAHL Complete (Ebscohost) | MJ derm* AND MJ tele* AND TX(guid* OR recommend*), Published Date: 20130101-20221231 | 11/23/2022 | 24 |
| Total (prior to de-duplication) |  |  | 839 |
| Total (after de-duplication) |  |  | 622 |

1. Grey Literature

| Organization | String | Search Date | Results |
| --- | --- | --- | --- |
| American Telemedicine Association <https://www.americantelemed.org/resource/learning-development/> | N/A | 11/23/2022 | 4 (manually added to EndNote, cannot access full text without membership) |
| American Academy of Dermatology <https://www.aad.org/> | Cannot search without membership |  | N/A |
| American Dermatological Association <https://ada1.org/> | N/A |  | 0 |
| U.S. Agency for Healthcare Research and Quality Evidence-based Practice Center Reports | “Telehealth dermatology guidelines” | 12/6/2022 | 4 |
|  | “Teledermatology guidelines” | 12/6/2022 | 0 |
|  | “Telemedicine dermatology guidelines” | 12/6/2022 | 1 |
| U.S. Agency for Healthcare Research and Quality Publications https://www.ahrq.gov/research/publications/search.html | “Telehealth dermatology guidelines” | 12/6/2022 | 0 |
|  | “Teledermatology guidelines” | 12/6/2022 | 0 |
|  | “Telemedicine dermatology guidelines” | 12/6/2022 | 0 |
